# Best Practices for Data Modernization Across the United States Public Health System: Scoping Review

**DOI:** 10.2196/70946

**Published:** 2025-10-24

**Authors:** Zebunnesa Zeba, Stella T Lartey, Polina Durneva, Shongkour Roy, Niharika Jha, Michael Arthur Ofori, Nidhi Mittal, Stella Dockery, Nichole Saulsberry Scarboro, Michelle Taylor, Ashish Joshi

**Affiliations:** 1 School of Public Health, University of Memphis, Robinson Hall, Memphis, TN, 38111, United States, 1 9012304821; 2 Department of Public Health Sciences, College of Behavioral, Social, and Health Sciences, Clemson University, Clemson, SC, United States; 3 Department of Information Systems and Business Analytics, Loyola Marymount University, Los Angeles, CA, United States; 4 Baltimore City Health Department, Baltimore, MD, United States

**Keywords:** health information system, health data integration, standard practices, data governance, data interoperability challenges, scoping review, PRISMA

## Abstract

**Background:**

The adoption of new technologies and data modernization approaches in public health aims to enhance the use of health data to inform decision-making and improve population health. However, public health departments struggle with legacy systems, siloed data, and privacy concerns, which hamper the adoption of new technology and data sharing with stakeholders. This paper maps how to address these shortcomings by identifying data modernization challenges, initiatives, and progress.

**Objective:**

This study aims to characterize evidence for data modernization–associated gaps and best practices in public health.

**Methods:**

This scoping review was conducted using the 5-stage framework developed by Arksey and O’Malley and was reported according to the PRISMA-ScR (Preferred Reporting Items for Systematic Reviews and Meta-Analyses Extension for Scoping Reviews) guidelines. A structured search was performed in the PubMed, Scopus, CINAHL, and PsycINFO databases and was complemented by a further search in the Google Scholar search engine, covering publications from January 1, 2019, to April 15, 2024. Eligible studies were peer-reviewed, published in English, and focused on data modernization initiatives within US public health system and reported on best practices, challenges, and outcomes. Search terms combined concepts such as “Data Modernization,” “Interoperability,” and “Public Health” using Boolean operators. Two reviewers independently screened titles, abstracts, and full texts using Rayyan QCRI, with conflicts resolved through consultation with a third reviewer. Data were extracted into Microsoft Excel and thematically analyzed.

**Results:**

This review analyzed 21 studies focused on public health data modernization. Across the literature, common components included transitioning to cloud-based systems, consolidating fragmented data into unified platforms, applying governance frameworks, and implementing analytics tools to support decision-making. Primary data sources were electronic health records, insurance claims, and disease surveillance registries. Key challenges identified across studies involved data quality issues, lack of interoperability, and limited resources, particularly in underfunded settings. Notable benefits included more timely and accessible data, improved integration across systems, and enhanced analytical capabilities, which collectively support more responsive and effective public health interventions when guided by clear standards and policy alignment.

**Conclusions:**

Progress hinges on balancing local adaptability with national coordination, improving data governance practices, and enhancing collaboration across institutions. These steps are vital to ensure that public health systems can deliver timely, accurate, and actionable information to support effective public health efforts.

## Introduction

### Background

The need to modernize public health data systems is more critical than ever. Data modernization goes beyond updating old technology; it fundamentally transforms how organizations manage, analyze, and leverage data. As the amount and complexity of data grow, outdated systems limit an organization’s ability to innovate and respond quickly to new challenges. Thus, continuous and accelerated efforts are crucial to fully realizing the vision of a learning health system [1]. By creating modern and secure data systems, organizations can not only enhance their operational efficiency but also better position themselves to adapt to future technological changes [2].

**Figure 1.**
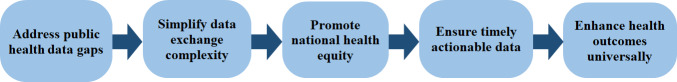
Strategic framework for advancing public health data systems.

Modernized data systems can significantly improve the accuracy and speed of data collection and analysis, leading to better health outcomes. In times of crisis, real-time data enable rapid interventions that can prevent adverse outcomes. Importantly, given the serious implications for population health and policy, this transformation plays a pivotal role in advancing public health outcomes (Figure 1). It is essential that our data systems are designed to be inclusive and equitable. By doing so, we ensure that public health initiatives not only reflect accurate data but also promote justice and positive change at the population level [3-6].

In response to the evolving landscape of public health challenges, the Centers for Disease Control and Prevention (CDC) stated that there is a pivotal shift toward enhancing data systems across US public health agencies [7]. The CDC has embarked on a comprehensive data modernization initiative, aimed at integrating advanced data analytics to effectively manage health-related data and improve the timeliness, accuracy, and utility of public health information systems [8,9]. As a result, the 2024 and 2025 milestones for the Public Health Data Strategy were crafted with contributions from over 100 national partners. These evolving milestones are closely monitored and updated to reflect ongoing progress, with success hinging on effective collaboration among state, tribal, local, and territorial public health departments, health care partners, and federal agencies [8].

There are many strategic initiatives on data modernization such as Health Level Seven International, Fast Healthcare Interoperability Resources, and the North Star Architecture, along with the efforts of the Office of the National Coordinator for Health Information Technology, which are pivotal in advancing public health data modernization in the United States [8,10-12]. To further enhance public health responses, continued improvements shaped by stakeholder feedback are essential. For these, the 2024‐2030 Federal Health IT Strategic Plan aims to promote health, enhance care delivery, accelerate innovation, and connect the health system through integrated health data [2].

The COVID-19 pandemic exposed stark vulnerabilities in our public health infrastructure, particularly regarding the obsolescence of data systems [13]. Throughout the crisis, health departments faced significant challenges in their data management capabilities, struggling with the collection, analysis, and dissemination of crucial information necessary for an effective response [14]. Often, different jurisdictions had inadequate and unreliable reporting, ambiguous timelines, difficulties in accessing data, and shifting interpretations from scientific institutions, making it challenging to leverage data for timely public health decisions. This situation underscored the critical need for a comprehensive modernization of public health data systems across the nation to ensure that data can be rapidly accessed, accurately analyzed, and effectively shared to better manage health emergencies and improve overall public health outcomes. While some papers have explored individual aspects of public health data systems, they are often limited to specific sectors or themes. This review offers a more comprehensive perspective by bringing together a wide range of data modernization efforts across the US public health landscape, aligned with national strategies and priorities. It addresses gaps in the existing literature and contributes meaningful insights for future research and practice. We have the mapping needed to mobilize health departments for seamless data sharing and state-of-the-art analytics. With investments in thoughtful data interoperability, governance, and training, public health can be brought into the modern data era. Ultimately, lives depend on having quality data in the right hands at the right time.

### Objectives

This scoping review aimed to address data modernization–associated challenges, initiatives, and progress in public health data systems that integrate advanced technologies and standardized practices. By identifying these challenges and best practices, the aim of this scoping review is to provide a comprehensive overview of the current state of public health data modernization, offering insights and recommendations to guide future efforts in creating a more efficient, equitable, and responsive public health infrastructure.

## Methods

### Search Period and Strategy

The search was conducted from January 10, 2024, to April 15, 2024, with no studies published after April 15, 2024, included in the review. The review was conducted in accordance with the 5-stage methodological framework developed by Arksey and O’Malley [15], which included (1) defining the research question specifically around challenges and best practices in data modernization; (2) identifying relevant research articles that discuss both successes and shortcomings; (3) selecting studies that provide comprehensive insights or innovative approaches; (4) organizing the data to highlight key themes and gaps; and (5) compiling, reporting, and summarizing the findings to outline clear paths forward and areas that need attention (Table 1).

**Table 1.** Inclusion and exclusion criteria

Category	Inclusion criteria	Exclusion criteria
Article type	Peer-reviewed original research articles	Editorials, commentaries, preprints, protocols, reviews, and conference abstracts
Language	Articles published in English	Articles published in other languages
Publication date	PPublished between January 1, 2019, and April 30, 2024. However, the database search concluded on April 15, 2024, and no studies published after that date were included in the review.	Published before January 1, 2019, or after April 30, 2024
Geographic focus	Studies conducted in the United States	Studies not conducted in the United States
Topic relevance	Title or abstract explicitly mentions “data modernization” or discusses conceptually related topics	No mention or conceptual relevance to public health data modernization in title or abstract
Public health focus	Focused on public health data systems	Not focused on public health data systems
Outcome evaluation	Includes outcome evaluation or insights into methods and implementation related to data modernization	Lacks outcome evaluation or implementation insights

The search strategy covered several bibliographic databases including PubMed, Scopus, CINAHL, and PsycINFO. Supplementary searches were also conducted using Google Scholar to identify additional peer-reviewed literature. The search terms used represent associated keyword combinations of public health, data modernization, and interoperability with appropriate Boolean operators (ie, “OR,” “AND”), as shown in Table 2. The process was documented in accordance with the PRISMA-ScR (Preferred Reporting Items for Systematic Reviews and Meta-Analyses Extension for Scoping Reviews) checklist, ensuring a rigorous and transparent review process (Checklist 1).

**Table 2.** Search strategy used in this scoping review.

Components	Review keywords
Population	Not required
Exposure/intervention	Intervention* OR program* OR policy* OR management* OR screening* OR detection* OR treatment* OR therapy* OR practice* OR guideline* OR service* OR support*
Comparator	Not required
Setting	“Public Health” OR “Public Health Administration” OR “Public Health Organization” OR “United States Public Health Service”
Outcome	“Data modernization” OR “Data Quality Management” OR “Data Curation” OR “Data Aggregation” OR “Data Integration”
Complete search strategy	((“Data modern*”[All Fields] OR “Data Quality Management”[All Fields] OR “Data Curation”[All Fields] OR “Data Aggregation”[All Fields] OR “Data Integration”[All Fields]) AND (“Public Health”[All Fields] OR “Public Health Administration”[All Fields] OR “Public Health Organization”[All Fields] OR “United states Public Health Service”[All Fields]))

### Eligibility Criteria

To keep the review focused, we applied predefined inclusion criteria during the title and abstract screening and full-text review phases. The criteria addressed article type, language, publication time frame, geographic focus, topical relevance to data modernization, public health focus, and outcome evaluation. These are summarized in Table 1.

### Screening Process

Articles identified from database searches and additional sources were reviewed using the cloud-based platform, Rayyan QCRI [16]. Following the removal of duplicate and inaccessible records (n=228), a total of 237 records proceeded to title and abstract screening. Two reviewer writers (ZZ and SR) independently screened these articles, labeled as “include,” “exclude,” or “may be with blinding enabled.” When there was a disagreement between reviewers, a third author was consulted to make the final decision. For articles not accessible through institutional holdings, the authors contacted the source author or journal to obtain the article. Finally, 56 articles were selected for subsequent review of the full article [17]. During this phase again, reviewers (ZZ and SR) independently assessed each of the full articles and discussed any uncertainties related to study selection [18].

### Data Extraction

The data were combined into a single spreadsheet in Microsoft Excel 2016 for checking and coding. The extraction form included the following categories: study objectives, manifestations of best practices, population of interest, framework/approach/action plan challenges and gaps in data modernization, proposed solutions, and approaches to addressing health inequities. To ensure clarity and comprehensiveness, the form was piloted on a sample of five articles by the research team. Data extraction for the remaining studies was conducted independently by two reviewers (ZZ and SR). The completed extraction tables were then reviewed collaboratively by both researchers. This process was iterative, allowing for refinement and ensuring that all relevant information necessary for thematic development was captured accurately.

### Quality Assessment, Collating, Summarizing, and Reporting Results

In a stepwise process, findings were reported following the PRISMA-ScR guidelines. This PRISMA-ScR flow diagram details the study selection process, number of studies screened, and those assessed for eligibility, as well as with reasons for inclusions and exclusions at each stage [19,20]. The collaborative review process among coauthors further enhanced the robustness of the results’ interpretation, ensuring a comprehensive and well-rounded analysis of the gathered evidence. Finally, the following information was extracted from each of the articles: author(s) name, year of publication, research objectives or question(s), method, and summary of findings of the theoretical framework about best practice, data modernization challenges, and gaps. From the included studies, we extracted and analyzed key information to identify emerging themes and categories related to public health data modernization. A general analysis was then conducted to classify the studies based on technical focus areas and public health domains. Particular attention was given to identifying commonly used best practices, which were synthesized and highlighted across the included literature. One of the central aims of this review was to extract and categorize the implementation challenges reported by researchers. These challenges were analyzed to provide insight into barriers that future initiatives may encounter. The extracted data for each thematic category, including best practices and challenges, were systematically organized and presented using summary tables.

## Results

### Overview

**Figure 2.**
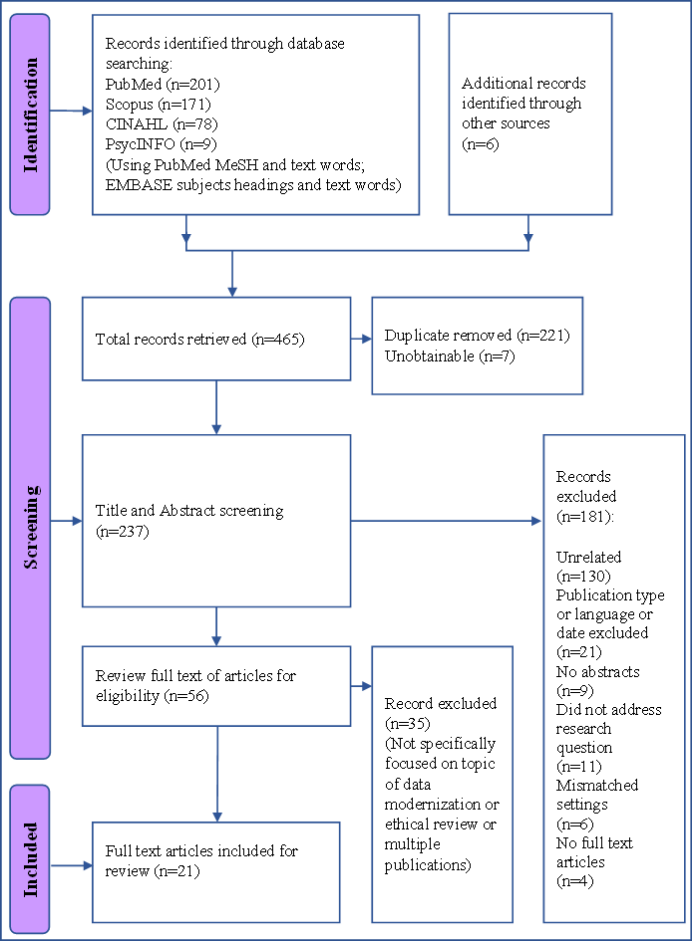
PRISMA-ScR diagram showing the flow of the search strategy and study selections. MeSH: Medical Subject Headings; PRISMA-ScR: Preferred Reporting Items for Systematic Reviews and Meta-Analyses Extension for Scoping Reviews.

This scoping review resulted in 21 papers on data modernization in US public health system and identified several key findings (Figure 2). Descriptive findings for each article are shown in Table 3. Seven studies utilized case studies or descriptive designs, primarily employing surveys, face-to-face interviews, and observations to collect data. Six studies applied mixed methods, integrating focus group discussions and interviews for a comprehensive perspective. Three studies used quantitative methods [21-23]. The remaining studies utilized a qualitative Delphi survey for gathering expert opinions and employed semistructured interviews to gather expert insights [24,25] (Table 4).

**Table 3.** Descriptive key findings of the included studies.

First author (year)	Objectives	Best practices	Data modernization challenges and gaps	Proposed solutions identified in studies	Addressing health inequities
Dore et al [9], 2022	The paper explores the creation of the LTC^a^ data cooperative to improve data infrastructure for public health	This paper focuses on integrating EHRs^b^ with LTC data to enhance data modernization and accessibility	The absence of a systematic EHR infrastructure for LTC, overlooked by initiatives like the HITECH^c^ Act, highlights existing gaps	The development of the LTC data cooperative represents a significant innovation in data integration	The integration of detailed clinical data aims to improve care and research for nursing home residents
Feldman et al [26], 2023	Maryland partnered with CRISP^d^ for COVID-19 contact tracing	Data enhancement enabled accurate records and timely public health responses	Integrated data and completeness were challenges	Workflows developed provide model for public health modernization	Enhanced data enabled outreach
Schmit et al [27], 2023	The study investigates the perceptions of state and local epidemiologists regarding increased federal access to data from the NSSP^e^	—^f^	Challenges include misinterpretation of data, lack of communication, privacy concerns, and current DUAs^g^ that restrict federal access, slowing emergency responses	Revise DUAs for flexible, secure data sharing	The paper does not explicitly address health inequities
Dignam et al [21], 2020	The study applies cumulative summary and Shewhart control chart methods to quickly identify significant shifts in childhood blood lead levels for timely public health action	Best practices include the application of control chart methods for real-time surveillance	Challenges include the need for continuous accurate data and the complexity of statistical models in routine surveillance	Enhanced surveillance methods are proposed to improve monitoring and management of child lead exposure	—
Facile et al [24], 2022	The paper discusses the application of CDISC^h^ standards to RWD^i^ to enhance data quality and interoperability across different health care and research settings	—	Significant barriers include the complexity of CDISC standards, lack of training, and the diverse nature of RWD sources	The paper suggests increasing training opportunities, improving tool availability, and enhancing collaboration between stakeholders	CDISC standards can reduce health disparities by ensuring equal representation in data analyses
Seidman et al [28], 2024	The main theme of the paper proposes strategies to build a nationwide health data ecosystem through regulation and funding	The authors highlight state health data utilities with federal guidance, model laws, and sustainable financing as key practices	They point to fragmented systems and inconsistent laws with limited funding as major barriers	Recommended approaches include federal guidance and sustainable funding	The study shows integrated systems improve equity in surveillance and vaccine delivery
Cocoros et al [29], 2021	The paper discusses RiskScape, an EHR-based web platform for public health data visualization	Use of automated EHR data for real-time public health surveillance. Flexibility to incorporate new conditions rapidly	EHR data varied in quality and coverage; staff needed training, support, and governance strategies to manage and interpret RiskScape data effectively	Standardized protocols, routine quality checks, and expanded site inclusion aimed to improve consistency	Risk-Scape enables the identification of inequities in disease burden. Supports targeted public health interventions to address disparities in health outcomes
Hohman et al [30], 2023	The paper describes the implementation, challenges, and lessons learned from the MENDS^j^	Standardize protocols, use open-source tools, and strengthen data governance	EHR data quality varied; extraction challenges, governance inconsistencies, and privacy concerns hindered reliability	Standardized protocols and governance improved chronic disease monitoring, public health response, and resource allocation through timely, integrated data using open-source tools	MENDS identifies disparities, offering chronic disease data to guide targeted interventions in underserved populations
Dobrinen et al [22], 2023	Describes COVID-19 surveillance methods used by RMTEC^k^ to support Tribal health departments in Montana and Wyoming	Multi-source surveillance, tribal data sovereignty	Tribal communities faced lack of access to real-time federal/state data, missing race/ethnicity data, fragmented surveillance systems, and delayed reporting	The paper suggests fully funding RMTEC to improve data management capabilities and establishing better education of federal and state agencies	The goal is to enhance data practices that respect tribal sovereignty and address American Indian/Alaska Native needs, reducing health disparities
Putnam-Hornstein et al [31], 2020	The paper mentions the integration of administrative records across California’s Health and Human Services programs to improve service delivery, policy development, and client-centered care	Establish standardized protocols for data extraction and integration. Use advanced data linkage techniques to accurately match records	Technical challenges in integrating data from various sources and formats. Ensuring data governance and addressing privacy concerns	Implementing standardized data integration protocols and using advanced data linkage techniques. Improved understanding of client needs and service utilization, leading to better-coordinated services	The integration of data helps identify and address health disparities by providing detailed insights into the SDH^l^ and targeted interventions to improve health outcomes for vulnerable populations
Bambekova et al [32], 2020	The paper explores PHATE^m^ integration of SDH and EHR data to improve care and public health surveillance	Geospatial tools and SDH-clinical integration support disparity reduction, with clinician training enabling effective data-driven risk assessment	Variability in data quality and completeness across different sources. Technical challenges in integrating SDH data with clinical records. Ensuring data governance and addressing privacy concerns	PHATE integrates SDH and clinical data via geospatial analysis, enabling targeted care, improved surveillance, and more effective resource allocation	Addressing disparities: PHATE helps identify areas with higher social risks and poorer health outcomes, supporting targeted interventions to reduce health disparities
López-Martínez et al [33], 2020	The main theme of the research paper is the development and implementation of a big data and machine learning platform to enhance medical decision support in population health management	Best practices highlighted in the paper include the use of big data technologies, advanced analytics, and machine learning	In this paper, the main challenges include complex data processing with inconsistent data quality	Leveraging big data and machine learning enhances interventions, diagnosis, and decision support, and reducing health care delivery costs	The research paper emphasizes the importance of utilizing a digital health platform based on machine learning and data integration principles to improve population health management
Dong et al [34], 2022	The main theme of the research paper is the development and operation of the JHU CSSE^n^ COVID-19 Dashboard to provide real-time surveillance report	The research emphasizes the importance of standardizing data collection, defining, and reporting practices across different locations to accurately assess the global epidemic situation	The study highlighted challenges in data visualization, reporting standardization, and data management decisions	The proposed solution involves adapting approaches for reporting data to improve the quality of real-time epidemiological data, emphasizing the need for consistent standards and practices across sources	The research paper does not explicitly discuss findings related to health equity in the context of the JHU CSSE COVID-19 Dashboard
Powell et al [35], 2023	The paper focuses on building a nationwide health data system in the United States, emphasizing regulatory and funding strategies to enhance data sharing and ensure equitable responses to health emergencies	Establish standardized regulations, state entities for data integration, innovative regulatory sandboxes, and adaptive strategies through ongoing evaluation	Key gaps include limited data disaggregation, inadequate demographic representation, and a lack of standardized public health reporting practices across regions	Promoting partnerships between public and private sectors, implementing inclusive data collection frameworks, and advancing legislation for equitable data practices	The paper highlights COVID-19–related disparities in health data and advocates for a comprehensive system to ensure equitable access to health information and services
Porter et al [3], 2023	The paper addresses the critical need to incorporate an explicit focus on structural racism within data modernization efforts to create equitable public health data systems	—	The paper outlines data modernization challenges: fragmented systems, varying regulations, limited funding, and incomplete EHR adoption	The proposed solutions include Federal Leadership and Guidance, funding Incentives and Standardizing health data practices across states	The paper addresses health inequities by examining the impact of structural racism on health data systems and suggesting ways to ensure fair data practices
Romero et al [36], 2021	The main theme of the research paper is to develop a reusable public health data analytics system to evaluate the impact of COVID-19 on populations served by health centers	Quality improvement focuses on optimizing data capture, flow, validation, and innovation across clinical and public health domains	Challenges included poorly aligned reporting requirements, lack of unique patient identifiers, and limited access to external data sources	The reusable public health data analytics system developed can be adapted and scaled for other health center networks to facilitate data aggregation, dashboards	The research paper addresses health equities by focusing on populations served by federally qualified health centers who are at increased risk for COVID-19 morbidity and mortality
Allen et al [23], 2024	The main theme of the research paper is to compare different EHR-derived hypertension prevalence measures with established survey data	Including vital statistics in EHR data improves accuracy and sensitivity, especially for subpopulations	Public health agencies struggle with limited authority, relying on incomplete voluntary reporting for chronic disease data collection	Using community Health Information Exchange networks supports chronic disease surveillance and addresses public health data reporting challenges	The study found higher prevalence in Black populations, highlighting the need for accurate, tailored data to address health disparities
Carney et al [7], 2023	The main theme of the research paper is to address the prevention and control of chronic diseases through a comprehensive strategy	The paper suggests that addressing data gaps in identifying populations with the greatest needs	Challenges include managing chronic disease complexity and meeting diverse data needs across health and social sectors	This paper focuses on updating outdated systems and improving surveillance to support healthier populations through coordinated data modernization efforts	The paper emphasizes the importance of reducing health inequities
Mangold et al [37], 2021	The main theme of the study is to assess the feasibility of collecting PGHD^o^ to enhance cancer registry surveillance	—	Challenges included recruitment, limited tech support, small samples, IRB^p^ delays, and PGHD development issues	Future efforts should enhance passive data collection technology, expand sample sizes, simplify IRB processes, and improve PGHD tools to better support observational research	Incorporating PGHD into cancer registry surveillance enhances treatment effects and quality of life, reducing disparities and improving care
Heacock et al [38], 2022	The paper aims to modernize environmental health data systems	Use of standardized methods, metadata, ontologies (standard vocabularies), and interoperable repositories	Overcoming data gaps, legacy systems, and workforce capacity limitations	Develop metadata standards, open formats, shared governance models (“data trusts”), artificial inteligenge and machine learning tools	Indirectly supports equity through open science, protecting tribal data, and inclusive data-sharing practices
Brody et al [25], 2023	Develop the best practices for emergency evidence searches	Use varied resources for comprehensive searches	—	Suggests transparency, centralized repositories, collaboration, and validated AI tools to enhance reliability	—

a LTC: long-term care.

b EHR: electronic health record.

c HITECH: Health Information Technology for Economic and Clinical Health.

d CRISP: Chesapeake Regional Information System for Our Patients.

e NSSP: National Syndromic Surveillance Program.

f Not applicable.

g DUA: data use agreement.

h CDISC: Clinical Data Interchange Standards Consortium.

i RWD: real-world data.

j MENDS: Multi-State EHR-Based Network for Disease Surveillance.

k RMTEC: Rocky Mountain Tribal Epidemiology Center.

l SDH: social determinants of health.

m PHATE: Population Health Assessment Engine.

n JHU CSSE: Johns Hopkins University Center for Systems Science and Engineering.

o PGHD: patient-generated health data.

p IRB: institutional review board.

**Table 4.** Study designs and methodology of the included articles.

Study design	Methods	Articles, n
Descriptive	Surveys and questionnaires Face-to-face interview In-depth interview Observation	7 [7,28,29,31,33,37,38]
Mixed (qualitative and quantitative)	Focus group discussion Face-to-face interview	6 [9,26,32,34,36,37]
Qualitative	Focus group discussion Literature review In-depth interview Key informant interviews Participatory research	2 [24,25]
Quantitative	Interview Retrospective analysis Cross-sectional study Cohort study	3 [21-23]
Others	Opinions Discussions Analytic essay Perspective essay	4 [3,27,30,35]

### Implementation Strategies and Best Practices

The review shows that modernizing public health data systems involves diverse strategies and best practices, which are categorized into 5 key areas: data enhancement, stakeholder engagement, surveillance and monitoring, data governance, and intervention and implementation, each emphasizing specific practices in public health, as shown in Tables3  5.

**Table 5.** Best practices application areas in public health data modernization.

Best practices application areas	Articles, n
Data enhancement	8 [7,23,26,33-36,38]
Stakeholder engagement	3 [9,25,37]
Surveillance and monitoring	8 [7,21,23,25,29,30,32,34]
Data governance	3 [30,31,38]
Intervention and implementation	2 [28,35]

### Data Enhancement

Data enhancement involves improving the quality, completeness, and accessibility of data, which is essential for effective public health decision-making. Carney et al [7] and Heacock et al [38] emphasize building infrastructure to support data sharing, while Feldman et al [26] highlight enhancement efforts in Maryland’s COVID-19 contact tracing system through enriched electronic laboratory reports incorporating demographic, vaccination, and hospitalization data—an initiative led by the Maryland Department of Health in collaboration with Chesapeake Regional Information System for Our Patients. Additionally, Romero et al [36] focus on public health analytics for COVID-19, and Dong et al [34] explore strategies for data integration and real-time sharing to support timely responses and informed decision-making. Consequently, Allen et al [23] highlighted data enhancement through standardized electronic health records (EHR) data extraction, open-source integration tools, and governance policies to improve chronic disease surveillance and address health disparities (Table 5).

### Stakeholder Engagement

Dore et al [9] and Brody et al [25] highlight the importance of stakeholder collaboration in enhancing public health data systems. Dore et al [9] focus on integrating EHR with Medicare claims through partnerships among providers, researchers, and agencies, while Bordy et al [25] emphasize the stakeholders’ role in improving data-sharing infrastructure for better pandemic response.

### Surveillance and Monitoring

Surveillance and monitoring systems described in these studies emphasize the use of EHR data and real-time tools to enhance public health efforts. For instance, Cocoros et al [29] explain how the RiskScape platform utilizes EHR data for near real-time chronic disease monitoring, enabling timely insights into population health trends. Similarly, Hohman et al [30] highlight the Multi-State EHR-Based Network for Disease Surveillance, which integrates EHR data from health exchanges to provide geospatial analysis and state-level disease tracking. Additionally, Allen et al [23] demonstrate the use of EHR-derived data to improve hypertension prevalence estimates, offering more precise local insights compared to traditional survey methods. Finally, Dong et al [34] describe how Johns Hopkins transitioned from Google spreadsheets to a real-time COVID-19 dashboard, using fusion logic to efficiently manage global pandemic data and improve decision-making (Table 5).

### Data Governance

Data governance in public health refers to a comprehensive framework of policies, roles, responsibilities, processes, and standards that determine who can access and use specific data, under what conditions, and for what purposes. It ensures data privacy, security, accountability, and transparency across systems and stakeholders. Standardization and protocols ensure consistency and interoperability across different public health data systems. According to Hohman et al [30], EHRs have been used to collect data from multiple health systems for real-time chronic disease surveillance. These protocols facilitated data integration, quality assessment, and visualization across varied formats, ensuring consistent data access and secure sharing. It applied statistical and geospatial methods to monitor and analyze chronic disease prevalence and trends across different populations. Related studies have discussed the implementation of standardized protocols, though these require proper referencing [30,31,38]. These studies reinforce the need for governance frameworks that adapt to technological advances while protecting individual privacy and enhancing data utility for public health action (Table 5).

### Intervention and Implementation

Intervention and implementation focuses on the practical application of data modernization strategies to improve public health outcomes. This category includes studies that discuss intervention strategies but often lack detailed reporting on their long-term outcomes and effectiveness, highlighting the need for more comprehensive evaluations (Table 5) [28,35].

### Data Modernization Challenges

As public health organizations work to modernize their data systems, they face a range of challenges that go beyond the technical issues that are summarized in Table 5. These challenges affect how data are collected, managed, and used, which in turn impacts the success of public health efforts. To build a strong and flexible public health data system that can handle both everyday needs and emergencies, it is important to understand these obstacles (Tables3  6).

**Table 6.** Data modernization challenges with included articles.

Challenges	Descriptions	Articles, n
Sociodemographic data gaps	Data collection: inconsistent methods across systems create incomplete datasets Demographic disparities: limited representation of underrepresented groups Recruitment: challenges with recruiting diverse participants due to resource limitations	5 [7,22,28,29,35]
Technical and operational challenges	Data reporting: continuous accurate reporting is hindered by technical limitations Real-time processing: processing large amounts of real-time data is resource-intensive Advanced models: difficulty implementing advanced models for predictive analysis	8 [7,21,22,30-33,37]
Integration and interoperability	Data integration: challenges in integrating data from various sources Data extraction: Difficulty in extracting data consistently from different platforms Privacy: managing privacy concerns while sharing sensitive health data	10 [7,23,26,28-30,34-37]
Data governance and privacy	Legal agreements: limitations in data-sharing agreements reduce data accessibility Staff training: inadequate training on data governance hinders effective data use Data siloing: siloed systems prevent efficient data exchange between organizations	10 [3,7,9,24,27,29-31,35,37]
Data quality and completeness	EHR^a^ variability: inconsistent quality across EHR systems Geographic coverage: incomplete geographic data limit comprehensive analysis Data linking: challenges in linking and de-duplicating patient data from multiple sources	5 [9,21,27,29,31]

a EHR: electronic health record.

### Sociodemographic Data Gaps

Gaps were noted in outdated data collection methods, insufficient workforce training, and fragmented governance structures. Sociodemographic data gaps present significant challenges in public health data modernization, particularly in accurately assessing disparities across different populations, which is reflected in other papers [7,22,28,29,35]. According to Dobrinen et al [22], the absence of detailed sociodemographic data prevents the Rocky Mountain Tribal Epidemiology Center from comprehensive monitoring and exacerbates health disparities during crises like COVID-19. Meanwhile, the study discusses the importance of establishing a nationwide health data ecosystem. One of the key strengths is its clear call for integration across state, local, and federal levels for improved public health data management, particularly in pandemic situations like COVID-19 [28]. Carney et al [7] highlight the lack of comprehensive sociodemographic data, particularly in identifying health disparities and a balance between data sharing and protecting privacy (Table 6).

### Technical and Operational Challenges

Technical and operational challenges are pervasive in the modernization of public health data systems. Dignam et al [21] highlighted the complexities involved in ensuring continuous, accurate data reporting and the challenges of implementing sophisticated statistical models for public health surveillance. Similarly, Bambekova et al [32] addressed technical challenges in integrating social determinants of health data with clinical records. López-Martínez et al [33] further underscored the operational difficulties associated with processing massive volumes of data in real time, which is essential for predicting clinical complications before they occur. The need for automation and operations in big data analytics platforms also presents significant technical hurdles (Table 6) [21,22,30].

### Gaps in Integration and Interoperability

Integration and interoperability of data from various sources are vital for a cohesive public health response. Feldman et al [26] addressed the challenges of integrating data across different platforms, ensuring data completeness and timeliness, which were crucial for the effectiveness of COVID-19 contact tracing. Hohman et al [30] further pointed out the technical challenges related to data extraction and transformation from various sources, emphasizing the need for consistent data governance and the protection of privacy. Similarly, aligning and integrating diverse datasets is particularly difficult when they originate from varying standards and formats (Table 6) [26,28,29].

### Barriers in Data Governance and Privacy

Data governance and privacy remain critical issues as public health systems integrate more data sources. In response, data modernization initiatives require both effective management change and strategic technology implementation. For instance, Schmit et al [27] highlighted the limitations imposed by existing data use agreements, which restrict federal access to essential data during public health emergencies, thus hampering effective responses. Putnam-Hornstein et al [31] emphasized the ongoing need for training and support for public health staff to ensure they can effectively manage and interpret integrated data, addressing both governance and privacy concerns. Additionally, several studies identified the challenges posed by restrictive data systems and inconsistent regulations, which limit the interoperability between public health and health care systems, limiting their usefulness for timely decision-making (Table 6) [27,29,30].

### Obstacle Between Data Quality and Completeness

The quality and completeness of data remain fundamental challenges in public health modernization efforts. Dore pointed out the necessity of a systematic EHR infrastructure, especially in long-term care settings, which had been previously neglected by initiatives such as the Health Information Technology for Economic and Clinical Health (HITECH) Act [9]. Similarly, Cocoros et al [29] emphasized the variability in data quality across different EHR systems, including issues with geographic coverage and the challenges of linking and deduplicating patient data from multiple care sites. These issues underline the importance of establishing robust systems to ensure comprehensive and high-quality data (Table 6) [9,21,27].

## Discussion

### Principal Findings

This scoping review identified a range of initiatives aimed at advancing public health data modernization in the United States, revealing both promising developments and persistent systemic challenges. While emerging efforts in areas such as EHR standardization, cross-sector collaboration, and real-time surveillance have shown potential [7,9,30,38], they remain limited in scale, inconsistently implemented, and often lack the infrastructure needed for long-term sustainability. In particular, barriers related to technical and operational capacity, integration and interoperability, data governance and privacy, and data quality and completeness continue to hinder progress across jurisdictions. The following discussion highlights modernized public health data systems that integrate advanced technologies and standardized practices, as well as the need for policy information to enhance data sharing and integration in public health systems. The COVID-19 pandemic has significantly accelerated data modernization efforts, presenting a unique opportunity to improve public health infrastructure. The primary challenge now is to sustain this thrust, ensuring efforts remain focused on the overarching goal of improving population health. In this context, the CDC’s strategic priorities, particularly accelerating data into action and managing change and governance, play a vital role [39]. Adopting this system-level perspective will be essential for achieving long-term success and maximizing the benefits of modernization initiatives [2,40].

These challenges faced in progressing data modernization are further elaborated in other studies. Across studies, recurring issues such as inconsistent data standards, fragmented infrastructures, and limited real-time access illustrate how structural weaknesses hinder effective and timely responses, particularly in tribal public health settings [22]. Moreover, we recommend standardized data integration and advanced linkage techniques to enhance the understanding of client needs and service utilization, thereby addressing health disparities [31]. Furthermore, the study underscores the pivotal role of big data and machine learning in strengthening public health interventions and decision-making, ultimately advancing health equity [33].

Although we did not find significant divergent views, the general perception in these reviewed studies highlights significant advancements and barriers in data modernization, emphasizing the importance of effective solutions and their impact on health equity. For instance, one of the studies revealed that collaboration between state health departments and health information exchanges to improve COVID-19 contact tracing served as a blueprint for future public health data modernization [26]. Another initiative focused on the development of a long-term care data cooperative to enhance care and research in nursing homes [9]. While not directly focused on health equity, this initiative supports an often underrepresented population in clinical studies [9].

Efforts to advance public health data modernization must build upon prior experiences in health care digitization. The HITECH Act, which catalyzed the widespread adoption of certified EHR, serves as a foundational example. While it successfully increased digital capacity, it also introduced new obstacles, including fragmented governance, limited interoperability, and system complexity that continue to hinder the seamless exchange of data across public health agencies [41].

A key insight from this review is the need to reform data use agreements. Existing agreements often limit data flow due to rigid language, security concerns, and a lack of adaptability across jurisdictions. Revising these agreements to support secure, timely, and flexible data sharing is essential for enabling rapid and coordinated public health responses, particularly in times of crisis [27]. Similar difficulties have been noted in other fields where the integration of data is essential. To limit its scientific and policy relevance, Kosnik et al [42] showed that integrated datasets may produce fragmented or inconsistent evidence if standardized reference and systematic validation are not used.

In addition to governance and legal frameworks, several studies emphasized the role of targeted surveillance tools in addressing environmental and health equity concerns. For instance, enhanced surveillance strategies for childhood lead exposure were shown to improve detection and response efforts in marginalized communities, where environmental risks are often concentrated [21]. These tools demonstrate how modernization can directly impact equity when tailored to specific population needs.

Standardization also emerged as a recurring theme in the literature. The adoption of Clinical Data Interchange Standards Consortium guidelines for real-world data was identified to ensure consistent and representative analysis, particularly for populations historically underrepresented in clinical trials. By standardizing data collection and analysis processes, these frameworks help mitigate biases and improve the inclusivity of health research [24].

Taken together, these findings reinforce the urgency of investing in core data infrastructure improvements. Achieving true interoperability across agencies, systems, and data sources requires transitioning to cloud-based platforms that support real-time automation, secure data access, and analytics on a scale. Without these foundational upgrades, the goals of equity, timeliness, and efficiency in public health modernization may remain out of reach [39,43,44].

From our scoping review, we identified that the challenge for public health is the decentralization of governance across 50 states [41]. These governance challenges are further compounded by shortcomings in demographic data collection, which restrict timely emergency responses and undermine equity in health outcomes [35]. In response, several studies advocate for stronger federal coordination and targeted funding mechanisms to support a more integrated, equitable data infrastructure that can address disparities amplified by the COVID-19 pandemic [28]. The literature also focuses on addressing structural racism in health data systems, proposing principles for equitable health data practices [3]. In parallel, targeted investments to strengthen analytic capacity in community health centers are essential, as timely and accurate data are critical for managing care among high-risk populations [36]. In response to these burning issues, public health policymakers must design a strategic approach that acknowledges the diverse starting points and capabilities across the nation, ensuring an inclusive and adaptable framework for data modernization best practices [41,43]. For this, now is the time for collaboration across jurisdictions to address common data challenges, with smaller or under-resourced health departments benefiting most from capacity building. Public health agencies should convene informatics staff to develop interoperable disease-agnostic systems that use common standards and technologies for efficient data integration.

The studies collectively underscore the need for targeted, equitable data modernization efforts to enhance public health interventions and address disparities. By implementing best practices such as standardized data models, secure data-sharing agreements, and advanced surveillance methods, public health agencies can significantly improve health outcomes and address inequities across various populations.

### Limitations

This study has some limitations. We limited our search to four databases—PubMed, Scopus, CINAHL, and PsycINFO—along with Google Scholar. This may have excluded relevant studies found in other databases. Many of the studies related to public health and data modernization were qualitative or uncategorized, which prevented us from conducting a meta-analysis. Additionally, we focused only on US-based articles, which may have excluded valuable insights from international studies. Lastly, only English-language papers were included, potentially missing important perspectives from non-English research.

### Conclusions and Recommendations

This scoping review highlights critical messages and interlinked issues in public health data modernization, including the impact on the modern data modernization initiative of the HITECH Act, the importance of adopting advanced technologies like North Star Cloud and Health Level Seven standards, and the ongoing challenges faced in the United States [11,12,45]. Studies reveal persistent issues with data visualization, reporting standardization, and real-time epidemiological data quality, essential for effective public health surveillance and response [40]. Despite advancements, we found that substantial gaps remain in population health informatics and informatics skills within the workforce in the field of data modernization. Governance issues, such as fragmented regulations and insufficient funding, complicate the development of a unified health data ecosystem. Misaligned reporting requirements, the absence of unique patient identifiers, and data gaps further hinder progress. Addressing these challenges requires robust policies and funding strategies to support standardized data collection, sharing, and utilization. Investing in data modernization, especially in informatics training and skill development, is crucial to fostering a more efficient, equitable, and responsive public health infrastructure.

## Supplementary material

10.2196/70946 Checklist 1 PRISMA-ScR checklist.

## Abbreviations

CDC: Centers for Disease Control and Prevention
EHR: electronic health record
HITECH: Health Information Technology for Economic and Clinical Health
PRISM-ScR: Preferred Reporting Items for Systematic Reviews and Meta-Analyses Extension for Scoping Reviews
