# Correction: Best Practices for Data Modernization Across the United States Public Health System: Scoping Review

**DOI:** 10.2196/87042

**Published:** 2025-11-11

**Authors:** Zebunnesa Zeba, Stella T Lartey, Polina Durneva, Shongkour Roy, Niharika Jha, Michael Arthur Ofori, Nidhi Mittal, Stella Dockery, Nichole Saulsberry Scarboro, Michelle Taylor, Ashish Joshi

**Affiliations:** 1 School of Public Health, University of Memphis, Robinson Hall, Memphis, TN, 38111, United States, 1 9012304821; 2 Department of Public Health Sciences, College of Behavioral, Social, and Health Sciences, Clemson University, Clemson, SC, United States; 3 Department of Information Systems and Business Analytics, Loyola Marymount University, Los Angeles, CA, United States; 4 Baltimore City Health Department, Baltimore, MD, United States

 In “Best Practices for Data Modernization Across the United States Public Health System: Scoping Review” [1], the authors made three corrections.

In the abstract, the search end date has been amended from “April 30, 2024,” to “April 15, 2024.” This has also been further specified in the “Methods” section and in Table 1.

In the abstract:

A structured search was performed in databases PubMed, Scopus, CINAHL, and PsycINFO, and was complemented by a further search in the Google Scholar search engine, covering publications from January 1, 2019, to April 30, 2024.

has been revised to:

A structured search was performed in the PubMed, Scopus, CINAHL, and PsycINFO databases and was complemented by a further search in the Google Scholar search engine, covering publications from January 1, 2019, to April 15, 2024.

In the “Methods” section:

The search was conducted from January 10, 2024, to April 15, 2024.

has been revised to:

The search was conducted from January 10, 2024, to April 15, 2024, with no studies published after April 15, 2024, included in the review.

In the “Publication date” row of Table 1:

Published between January 1, 2019, and April 30, 2024

has been revised to:

Published between January 1, 2019, and April 30, 2024. However, the database search concluded on April 15, 2024, and no studies published after that date were included in the review.

These corrections will appear in the online version of “Best Practices for Data Modernization Across the United States Public Health System: Scoping Review” [1] on the JMIR Publications website, together with the publication of this correction notice. Because these changes were made after submission to PubMed, PubMed Central, and other full-text repositories, the corrected article has also been resubmitted to those repositories.
